# The replication timing program in the hands of two HDACs

**DOI:** 10.15698/mic2014.08.163

**Published:** 2014-07-25

**Authors:** Kazumasa Yoshida, Armelle Lengronne, Philippe Pasero

**Affiliations:** 1 Equipe Labellisée Ligue Contre le Cancer, Institute of Human Genetics, UPR 1142, CNRS, 141 rue de la Cardonille, 34396 Montpellier, France.; 2 Department of Cellular Biochemistry, Graduate School of Pharmaceutical Sciences, Kyushu University, 3-1-1 Maidashi, Higashi-ku, Fukuoka 812-8582, Japan.

**Keywords:** DNA replication, epigenetics, histone deacetylases, budding yeast

## Abstract

In eukaryotes, duplication of genomic information depends on the sequential activation of multiple replication origins distributed along the chromosomes. Replication origins differ in initiation time, chromatin structure and three-dimensional position in the nucleus. Recently, we have performed a systematic analysis of the role of histone deacetylases (HDACs) in the regulation of origin activity in budding yeast. We have found that the epigenetic regulation of repetitive sequences is a key determinant of the DNA replication program. Indeed, our study revealed that two histone deacetylases, Rpd3 and Sir2, have opposite effects on the replication timing program. Rpd3 delays initiation at late origins, whereas Sir2 promotes efficient activation of early origins. Remarkably, we also found that Rpd3 and Sir2 regulate initiation at ~200 replication origins located within the ribosomal DNA (rDNA) array. We propose that this epigenetic regulation of repetitive origins controls the replication timing program by modulating the availability of limiting initiation factors.

Faithful replication of chromosomal DNA is essential for the maintenance of genome integrity. The timing and the efficiency of origin firing is flexible and is finely tuned during development and differentiation. Although the physiological significance of the replication timing program remains unclear, it is generally believed that it coordinates DNA replication with transcription, repair and other cellular functions. A large body of evidence indicates that the replication timing program is determined by a combination of local and global mechanisms that affect the structure of chromatin and the three-dimensional organization of the nucleus. These epigenetic modifications modulate the licensing of origins and/or their accessibility to initiation factors. The abundance of initiation factors restricts the number of origins that can be activated simultaneously. These factors are recycled from early to late origins during S phase.

The histone deacetylase Rpd3 has been implicated in the formation of inhibitory chromatin structures that delay initiation at a large fraction of origins. Whether Rpd3 acts specifically on late origins or whether it also represses early origins is currently unclear. Besides Rpd3, it has been reported that the Sir2 and Hst1 sirtuins regulate initiation at a subset of origins in an opposite manner. However, the mechanism by which HDACs modulate the execution of the replication timing program in budding yeast remains poorly understood.

To address the effect of HDACs on DNA replication, we have generated a collection of deletion mutants for all the yeast HDACs and monitored the effect of these deletions on the origin usage by BrdU-IP-chip and BrdU-IP-seq. In these assays, newly replicated DNA is labeled with BrdU, immunoprecipitated (BrdU-IP) and hybridized on high-resolution tilling arrays (chip) or sequenced (seq). Among the ten HDAC mutants, only two showed significant effects on replication profiles. As reported previously, a large fraction of origins were activated prematurely in *rpd3*∆ cells. These modifications correlated with local changes in acetylation profiles, but were independent of the time of origin activation. In contrast, BrdU incorporation was globally decreased in the *sir2*∆ mutant relative to wild type cells. We verified that these modifications were not due to changes in the entry into S phase, nucleotide pools or replication checkpoint response. These data suggest that Rpd3 and Sir2 are major regulators of the temporal regulation of DNA replication.

About a third of yeast replication origins are located within repetitive sequences, such as the rDNA array. This locus consists of 100~200 copies of a 9.1 kb repeat containing a replication origin, but only 20% of these origins fire within a given S phase. Active origins are located downstream of transcriptionally active gene and form clusters, separated from each other with inactive regions repressed by Sir2. It has been recently reported that rDNA origins are able to compete with single-copy origins for initiation factors. We therefore reasoned that Sir2 could control the activation of single-copy origins by modulating the ability of rDNA origins to titrate limiting initiation factors.

To address this possibility, we have monitored rDNA replication using quantitative PCR. This analysis revealed an inverse correlation between initiation rates at repetitive and single-copy origins in* sir2*∆ cells. Moreover, a reduction of the rDNA array to 20 copies suppressed the effect of the *SIR2* deletion on single-copy origins, which is consistent with our model. Finally, we found that deletion of the *SIR2* gene increased H4K16 acetylation not only at the rDNA origin, but also at the replication fork barrier (RFB), a sequence involved in the formation of extrachromosomal rDNA circles (ERCs). Since ERCs contain replication origins, they could also titrate limiting initiation factors in *sir2*∆ cells.

We next analyzed activity of rDNA origins in *rpd3*∆ cells. In contrast to the premature activation of single-copy origins, rDNA replication and ERC levels were significantly decreased in the *rpd3*∆ mutant. Moreover, we found that the reduction of rDNA replication in the *rpd3*∆ mutant depends on Sir2. Indeed, *SIR2 *deletion in *rpd3*∆ cells restored a normal replication profile, both at the rDNA locus and at single-copy sequences. Finally, to confirm that rDNA origins compete with single-copy origins for limiting initiation factors, we overexpressed the initiation factors Sld3, Sld7 and Cdc45 in *sir2*∆ cells. Remarkably, this overexpression suppressed initiation defects at early origins.

Altogether, these data indicate that the modulation of rDNA origins and the formation of ERCs are key determinants of the replication timing program in budding yeast. We propose that the histone deacetylases Sir2 and Rpd3 modulate the ability of rDNA origins to sequester limiting initiation factors (Fig.1). These data are consistent with the fact that local acetylation levels do not correlate with the timing of origin activation at the genome-wide level, even though they strongly affect BrdU incorporation at a large fraction of origins in *rpd3*∆ cells. Interestingly, it has been reported that rDNA origin activity and the accumulation of ERCs is associated with aging in yeast. It is therefore tempting to speculate that the decreased origin usage observed in *sir2*∆ cells recapitulates a process occurring in old cells. Another important issue is whether HDACs also control the replication program in other organisms through the regulation of initiation at repetitive DNA. The human genome contains a large amount of repetitive sequences in heterochromatin. Human cells may also control the replication of these repetitive sequences to prevent unnecessary competition with single-copy origins, as it is the case in budding yeast. Future studies will undoubtedly provide new insights into the possible role of HDACs and repetitive sequences in the regulation of complex replication program in higher eukaryotes.

**Figure 1:**
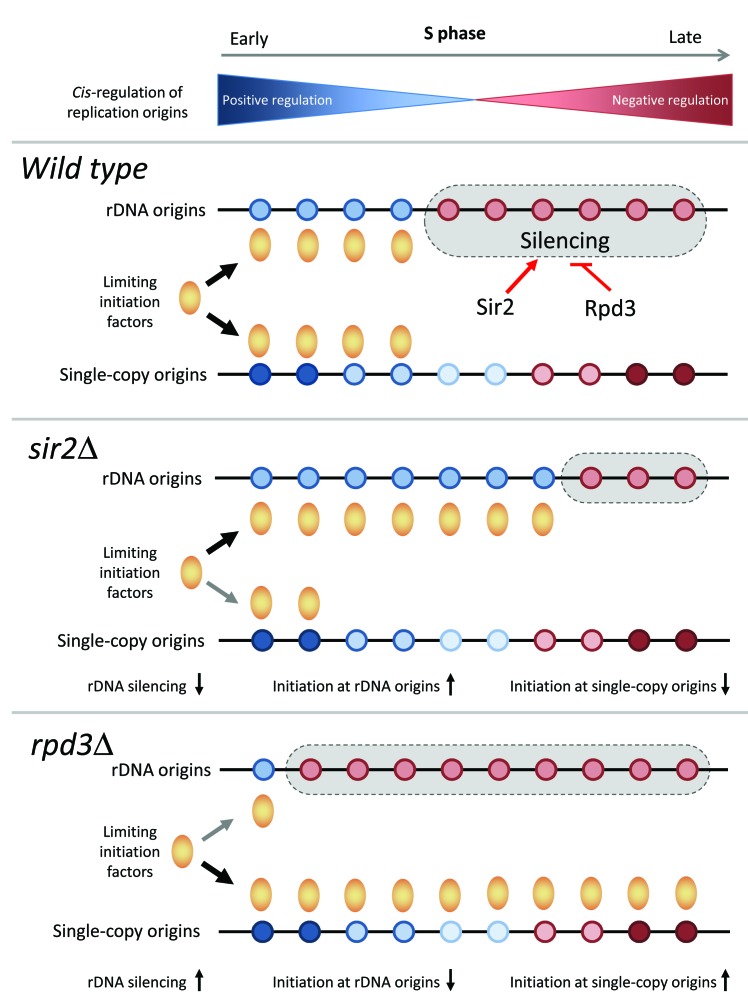
FIGURE 1: Regulation of replication timing program in budding yeast by the Sir2 and Rpd3 histone deacetylases. See main text for details.

